# Kali Iodatum, Etc., in Phthisis

**Published:** 1883-10

**Authors:** E. W. Berridge

**Affiliations:** London


### CLINICAL BUREAU.

### KALI IODATUM, ETC., IN PHTHISIS.

E. W. Berridge, M. D.

December 18th, 1872, Miss D., æt. 22, consulted me. Her father and brother both died of phthisis. She had pneumonia the last two winters. Her present symptoms are as follows: Left chest dull on percussion, with bronchial breathing there; sneezing; running from nose; loss of taste and smell; soreness of chest; no appetite; weakness; nose feels sore; cough with thick expectoration, the cough causing pain in left side as if something were lifted up and down; for a week cough has been worse when lying on left side; for the last fourteen days, at times, has had *shooting pains from centre of chest to front of left shoulder* when coughing; had the same two years ago. The italicized symptom was evidently the most characteristic, the most peculiar, and the one which most individualized the case from others of the same disease. At p. 11 of vol. 2 of *Gregg's Homœopathic Quarterly* (the Illustrated Report, a portion of which has been subsequently republished, and is an indispensable work to the homœopathician) the following symptom is attributed to *Kali iodatum*, "violent stitches in middle of sternum, extending to the shoulder." The particular shoulder is not defined, but in this case the left shoulder was affected, and if my memory serves me, Dr. Gregg told me that he had cured the sore pains when going to the right shoulder. [*Allen's Encyclopædia* erroneously gives "shoulders" instead of "shoulder;" the same mistake occurs on p. 1012 of his *Index*, under "Middle of Sternum." At p. 247 the rubric, "Middle Sternum," seems repeated as a subregion of "chest," with a different list of symptoms and remedies. I will also here point out that in symptom 380 of *Moschus* "ease" should be read "worse," and the symptom corrected thus at p. 700 of *Index*. Symptom 11 of *Vipera lachesis bupcephalus fel*, should read "external melleolus" instead of "internal," and the corresponding correction made at p. 82 of *Index*. In *Plantago*, symptom 83, p. 556, line 4, for "hand" read "head," and in symptom 349 for "purgings" read "prurigo," and correct in *Index* accordingly.] This medicine being the only one known to produce this symptom, and the other symptoms being fairly covered by it, I dissolved four globules of Jenichen's 1m in water, and gave a spoonful every four hours.

Dec. 19th.—Shooting gone; very little sneezing; loss of smell, coryza, soreness of chest and nose all better; slept better; to-day has the pain in left side only when lying on it; cough better; has palpitation when walking; less bronchial breathing. Stop medicine.

Dec. 20th.—Catarrh gone; last night the shooting on coughing was bad, but has not returned to-day; less cough or soreness of chest; no palpitation; feels stronger.

Dec. 21st.—Much better; taste and smell returned; cough very much better last night and to-day; no pains in chest; no pains in side for two days; no soreness of nose or sneezing; feels better.

Dec. 23d.—Much better, and has been out-of-doors; cough less, not so much aggravated by lying on left side; feels stronger and sleeps well; less bronchial breathing; no other symptoms.

Dec. 24th.—Cough worse; *pain in left hypochondrium on coughing, or on deep inspiration, or on lying on sides, especially the left, or on walking much; when coughing, shooting upward in left hypochondrium;* oppression of chest with the cough; yesterday some palpitation. The italicized symptoms led me to *Belladonna* (see Dr. Simmons' *Cough Repertory*, now out of print, but incorporated in that being published in HOMŒOPATHIC PHYSICIAN), and I gave a dose of 60m (Fincke) in water three times a day.

Dec. 27th.—The pain in hypochondrium disappeared the following night; cough much less; lies better on left side; appetite much better, much stronger; took a long walk yesterday; sleeps well; no palpitation; chest feels easier; less bronchial breathing. Stop medicine.

1873, Jan. 3d.—Has steadily improved; cough much better; for the last five days has been able to lie on left side without bringing it on; no return of pains in chest or hypochondrium; breathing easier; appetite good, but does not care for meat; much stronger; auscultation more natural.

Jan. 6th.—Cough not so well for two days; it is paroxysmal, excited by a feeling of choking in throat as if she could not get her breath; last night cough was worse from lying on left side; to-day dull pains in left side; cough relieved by drinking cold water; rather more bronchial breathing in left chest. Cough relieved by cold water is found under *Caps.*, *Caust.*, *Cupr.*, *Sulph.* (the last is Dr. Fincke's clinical observation verified by this case.)

Of these four medicines only *Sulph.* has aggravation of cough from lying on sides (*left* side not yet observed), and as it also has a marked action on the *left* lung, I selected it and gave Cm (Fincke) in water three times a day.

Jan. 7th.—Cough better; sleep not good last night from dyspnœa, which was worse when lying on right side (*Sulphur* symptom); no choking; less pain in side; coughs during sleep; can walk faster than formerly. Stop medicine.

Jan. 8th.—Cough and dyspnœa much less; slept well; no pain in side; appetite better; stronger; no palpitation; less bronchial breathing.

Jan. 9th.—Much better, but a little pain in side. (Weather wet.)

Jan. 16th.—Cough gone for two or three days; still has dyspnœa on ascending, but at no other time; much stronger; no pain; sleeps well; appetite good, and eats meat; yesterday had sharp pain in left hypochondrium when ascending; still some bronchial breathing.

April 17th.—Has remained quite well, running and being exposed to cold and wet with impunity, till the last six days, when she has felt weak; to-day she fainted; dull headache. Took six doses of *China*$^{cm}$ (Fincke) which soon relieved her.

Oct. 2d.—Has remained quite well till September 27th, when she caught cold. Now has cough worse in morning; thick, blackish expectoration with cough; cough causes pain in left hypochondrium and nausea; palpitation returned; weak feeling at times, coming and going suddenly; bronchial breathing of left chest increased, with a little crepitation there.

Black expectoration is found under *Arn.*, *Bell.*, *Chin.*, *Kali bichr.*, *Lyc.*, *Nux*, *Oxal. ac.*, *Rhus.*

Of these, *Arn.*, *Bell.*, *Lyc.*, *Nux*, have pain in hypochondrium on coughing; and of these four, *Nux* has nausea with cough. I gave one dose of *Nux vom.*$^{mm}$ (Bœricke).

1874, May.—Reports that she has remained quite well, except at times a pain in side.

1876, July 31st.—Have not had to prescribe for her since. She is in good health, is married, and has a healthy child.

1882.—Still remains free from her old phthisical symptoms.

*Comments:* In the above case, each remedy was selected according to the symptoms only, without regard to the name of the disease or any pathological theories, and the result left nothing to be desired. A *professed* homœopathic physician (who before he died saw the error of his ways) once asked me what remedy I gave in the first stage of phthisis, and seemed considerably surprised when I told him that I gave whatever remedy was indicated by the symptoms. How would a pathological-prescriber have treated this case, and what success would his miserable routine treatment have procured? What would pathology and physiology have availed him in the selection of the remedy? Will any of these pseudo-scientists inform us what is the pathological signification of the symptom "shooting from centre of sternum to shoulder"? Will they tell us with what particular change of lung tissue this symptom is necessarily associated, and what different lesions would have existed had the pain gone in the opposite direction? If they cannot do this, what becomes of their boasted "progress of pathological discovery," and "improvements on Hahnemann's Homœopathy"? It may be said that this knowledge is in the future. Be it so, but when reached, will it enable us to select the remedy and to cure with any more certainty than our semiological researches do at present? And if not, what is the practical value of it, so far as therapeutics, as distinct from hygiene, is concerned? The truth is, that symptoms which are diagnostic of the disease are rarely, if ever, diagnostic of the remedy; the reason being, that the symptoms diagnostic of the disease are those common to a very large number of cases, which we are thus enabled to group under a generic name; whereas the symptoms diagnostic of the remedy are those which individualize one case of the same disease from another. The former symptoms are, moreover, found under a large number of remedies, provided it has been practicable to push the provings so far, the latter only to a few remedies; hence the former are not characteristic, the latter are.

